# Monthly Summary

**Published:** 1897-03

**Authors:** 


### Monthly Summary.

DIAGNOSIS OF PULPITIS.—By Dr. J. N. Crouse.—I do not think the essayist has touched upon the vital points of pulpitis. The important point is to diagnose the case. Pulpitis is the first stage of a dying pulp, and it is frequently difficult to locate. I allude to a case in which a number of teeth are filled, in any one of which you might suspect the pulp to die. The patient will have severe paroxysms of pain, hard to locate and increasing in intensity

What are the symptoms of pulpitis, and how are you going to tell them? I have been able to locate pulpitis in one of two or three ways. My first method is to tap on the teeth and see which one showed tenderness, for, if you have pulpitis you usually have more or less tenderness on tapping. Then you can make an investigation and see if your diagnosis is correct. If so, you can isolate that tooth by putting the dam over it and apply cold water to test it for sensitiveness. If that increases the pain apply a heated instrument to the tooth. If pulpitis be present the patient will have an extreme paroxysm of pain. As soon as I found that I was on the right tooth I should drill in there and expose the pulp as quickly as possible, because it is only a question of a few days when the pulp will die. Of course, it may die and the pain pass away if you do not drill into the tooth, and the patient may not suffer enough to demand on operation. I think there are cases where the pain passes off when the pulp is dead. But acute pulpitis is the first stage of death, and I would relieve the pain as quickly as possible. If there is any place I could reach with a cataphoretic apparatus, I would reach it.

Just before I started to attend this meeting a patient came to my office. He was suffering very much with toothache and had been in pain for two or three days. Some time ago I treated him for a severe case of abscess of the antrum, and ever since he has located pain in the antrum. He knows where it is and is positive about it. I told him I did not care where he located the pain, but to tell me what his symptoms were, and he replied that he had sharp paroxysms of pain. I began to tap on a lateral incisor, and found that this was the tooth which had given trouble for three days. I drilled into it, passed a broach two-thirds of the way up quickly, secured free bleeding, and the patient was relieved. He had been suffering severely and I did not want him to suffer any more. After drilling in I applied some carbolic acid, partially killed the pulp, cured the pulpitis, and I expect to treat the cavity tomorrow morning.—Proceedings of Chicago Dental Society, "Western Dental Journal,"

---

NITRATE OF SILVER.—Dr. A. W. Harlan exhibited specimens of teeth treated with nitrate of silver.

Dr. A. W. Harlan: At the last meeting of the American Dental Association, held at Saratoga, Dr. Bethel, of Kent, Ohio, read a paper on "The Use of Nitrate of Silver for Filling Minute Root-Canals by the Method of Cataphoresis." He made solutions of 10, 20, 30, 40 and other per cents of nitrate of silver and drove these into the roots by the cataphoretic instrument, In the discussion that took place, which has been published in the "Dental Cosmos" and "Ohio Dental Journal," and possibly one or two other journals, the statement was made by one speaker that he was afraid this would cause complete or partial discoloration of the tooth. Among others who took part in the discussion was your present speaker. I denied that this would sensibly discolor the interior of a tooth from numerous experiments that I had made, and finally the question narrowed itself down to one which was stated by Professor Pruman to be a question of veracity. I did not reply to that, as I think personalities in scientific debate are entirely unnecessary.

I hold in my hand a number of specimens that have been treated with nitrate of silver, not by the cataphoretic process, but by ordinary processes of making solutions from 10 per cent. up to a saturated solution and using freshly extracted teeth (in the middle row on this card), to be dropped into these solutions and allowed to remain for from three to five or six days; then taking them out, simply washing them, and making a cut on the surface of the tooth, which you will see. On the first row in this card there are four roots that have been filled, the interiors of which roots were first treated with from 30 to 50 per cent. solutions of nitrate of silver and allowed to remain twenty-four, forty-eight, seventy-two, and ninety-six hours, and without cleaning the roots out or drying them, the root fillings were forced in and the teeth cut by Dr. Wikoff. They were all planted in plaster of Paris, so that the roots could not be seen. One in particular, a molar tooth, it was the only molar the roots of which had been filled with an almost saturated solution of nitrate of silver on Saturday morning and allowed to remain until Tuesday afternoon, and cut down by Dr. Wikoff this morning. These specimens speak for themselves. You can see on the reverse side the intense blackening of the cementum, and on the specimens of root fillings, one of which has been filled with amalgam with the white solution of nitrate of silver, is another. After a lapse of three months or a little more it does not show appreciable discoloration. I have one specimen more than three years old, and I have other specimens which were made in September, 1894. I pass them around simply in the interest of accuracy of observation. These teeth were all moist. Those not freshly extracted were previously soaked in water for days, so that they became thoroughly moistened.

One of the specimens I made was kept in moisture and stayed there between two and three months, being looked after by my son. He wanted to fill the root of that tooth, and it was kept in a jar to see whether any change took place. I am not advocating the fillings of roots of teeth with nitrate of silver. I simply show the results of the silver on the enamel, dentine and cementum, and under the circumstances I have related nitrate of silver, as you know, as soon as it parts with its oxygen, ceases to be nitrate of silver and deposits the oxide, which in time is converted into metallic silver, not wholly, but partially. Oxide of silver is not a penetrating agent. Nitrate of silver, as any one would know from looking at a photographer's fingers, is an agent that is self-limiting—that is, it will penetrate a certain distance and then stop on account of the formation of the oxide, which prevents its further penetration.—Proceedings of Chicago Dental Society in "Western Dental Journal."

---

PULPITIS VS. PERIDONTITIS.—I may have misunderstood Dr. Davis as making the statement that in tapping a tooth there was never any soreness or tenderness where there was pulpitis. It has been my impression in a number of cases, before operating, that the tooth involved had a dead pulp, thinking that the accompanying tenderness indicated it; and I would find upon operating that it was a case of deep-seated pulpitis. Such cases I believe have been spoken of by Dr. Black, who is an authority on this subject. Dr. Black says, If I am not mistaken, that 'in cases of deep-seated pulpitis, there is always marked tenderness, which is often confused with apical pericementitis following the death of the pulp."—Dr. Chas. J. Merriman in Chicago Dental Society.—*Western Dental Journal.*

---

NUCLEIN IN DENTAL PRACTICE.—We have been using for a few months past a solution of Nuclein prepared by Parke, Davis & Co., from the formula of Prof. Vaughn, in slugish abscesses, and pyorrhea pockets. So far the results seem satisfactory and the action of this natural germicide seems very kindly. It is especially indicated where irritation or destruction of embryonal tissue—so often following the application of the usual germicides—is feared, as it is supposed to make war only upon pathogenic bacteria and does not interfere with the healthy tissues.—Editorial, *Western Dental Journal*

PULPITIS.—This matter of pulpitis is one that gave me a great deal of nervous disturbance in my earlier practice. There was no condition which presented itself to me at that time that made me think more, until I began to control it. When the dentist has a severe case of pulpitis with extreme pain he feels as helpless as at any time in his practice. I have learned somewhat how to treat pulpitis. I have found by experience and from information gathered from different sources, that the only way to treat pulpitis and to relieve the pain is to expose the pulp and let out some blood. Dr. Davis said that his treatment for pulpitis is arsenic. I should disagree with him. I think there are cases where it is necessary to preserve the pulp for some time. This is true with the anterior teeth of children, where the pulps are nearly exposed, and it is necessary to preserve them for some time. The tooth should be restored to its normal condition as nearly as possible in order that its full development may be attained.

The discovery of cataphoresis has made it possible for the dentist to allay the pain of pulpitis as nothing else has. I cannot agree with the essayist, however, that we can destroy a pulp successfully by cataphoresis. I do not believe that as a pulp-destroyer, cataphoresis is a success. I have no new remedies or other procedures to offer.—Dr. J. P. Kester, in Proceedings of Chicago Dental Society.—"Western Dental Journal."

---

CHLOROFORM is a more satisfactory anesthetic than ether for operations liable to be complicated by difficult or suspended respiration, in those cases it is reasonably safe, and when carefully administered, it may be confidently recommeded to the profession for this particular kind of surgical work.—Gay.

---

"CATAPHORESIS."—In sensitive dentine, all that can be obtained with this process can in the same time be accomplished with hot alcohol, hot air, and the usual old orthodox methods.—"Western Dental Journal."

"MURDER (AND OTHER CRIMES) WILL OUT."—The solemn text, "Be sure your sin will find you out," had a strange, almost ludicrous, illustration in Pittsburg a short time ago. The house of Police Surgeon R. L. Taylor was entered by a burglar. The doctor, however, frightened him away. He caught but a glimpse of the man and could only tell that he was a Negro. Searching the premises he found that the thief had taken nothing away, but in ransacking the kitchen for the silverware he had found a piece of pumpkin pie and took a bite of it, leaving a clear impression of his teeth. Dr Taylor made a plaster cast of the tooth prints. The next day a Negro was arrested on suspicion, and his teeth corresponded exactly with the mold. When he was confronted with this evidence he made no defense.

---

A GOOD PRESCRIPTION.—When you have labored and perspired over the tiresome and seemingly insurmountable difficulties attending some difficult dental operation, and your hands and brain are "fagged" out—a good prescription is to discharge the patient and try again at another sitting. A good night's rest following attendance at the theatre will have given you new capabilities to overcome difficulties, and on the morrow you will be able to bring to a successful conclusion some work which the day before seemed impossible. Try this prescription.—*Western Dental Journal.*

---

WHOOPING COUGH BACILLUS.—Kurlov has been investigating the saliva of whooping cough patients, and has found in every case and in them alone, a certain special, spore-forming, ciliated amœba, which he suggests may be the cause of the disease.—"Bulletin Medical."

---

AN INDIAN HAS A TOOTH FILLED.—An Indian who had a tooth filled and another pulled at Waterville, Me., furnished the first instance of a red man patronizing a dentist which had come to the knowledge of a practitioner of thirty years in that place.—"Chicago Tribune."

